# Electroacupuncture Pretreatment as a Novel Avenue to Protect Heart against Ischemia and Reperfusion Injury

**DOI:** 10.1155/2020/9786482

**Published:** 2020-05-18

**Authors:** Jiyao Zhang, Luwen Zhu, Hongyu Li, Qiang Tang

**Affiliations:** ^1^Graduate School, Heilongjiang University of Chinese Medicine, 24 Heping Road, Xiangfang District, Harbin 150040, Heilongjiang, China; ^2^Brain Function and Neurorehabilitation Laboratory, Second Affiliated Hospital of Heilongjiang University of Chinese Medicine, 411 Guogeli Street, Nangang District, Harbin 150001, Heilongjiang, China; ^3^Rehabilitation Center, Second Affiliated Hospital of Heilongjiang University of Chinese Medicine, 411 Guogeli Street, Nangang District, Harbin 150001, Heilongjiang, China

## Abstract

In recent years, the efficacy of electroacupuncture (EA) pretreatment generating ischemic tolerance mimicking ischemic pretreatment (IP) has been continuously confirmed, which was first found in the brain and then in the heart. Furthermore, researchers have observed the intensive cardioprotection impact of EA pretreatment on patients undergoing percutaneous coronary intervention (PCI) and heart valve replacement, indicating that EA pretreatment tends to be a valuable and advantageous avenue for preventing acute myocardial ischemia/reperfusion (I/R) injury or treatment of ischemic heart disease (IHD). In reality, the heart protection mechanism of EA pretreatment is robust and pleiotropic, of which the regulatory molecular pathways are involved in multichannel, multilevel, and multitarget, including energy metabolism, inflammatory response, calcium overload, oxidative stress, autophagy, and apoptosis. Through a growing number of clinical tests and basic experiments with animal models, researchers progressively explored the optimal acupoints and parameters, where EA pretreatment induced acute and delayed ischemic tolerance for myocardial protection. Thereby, this article aims to collect the relevant evidence on EA pretreatment against myocardial ischemia/reperfusion injury (MIRI) and summarize the mechanism of cardioprotection of EA pretreatment to provide ideas and methods for further clinical applications.

## 1. Introduction

According to statistics, more than 8.5 million people throughout the world die from acute myocardial infarction (MI) each year [1, 2], of which limited therapeutic options are currently available against ischemic myocardial injury. At present, the medical treatment principle for ischemic heart disease (IHD) is to restore the blood perfusion for the ischemia myocardium [3, 4]. However, percutaneous coronary intervention (PCI), coronary artery bypass grafting (CABG), percutaneous transluminal coronary angioplasty (PTCA), emergency thrombolysis, or other therapy methods can restore the blood flow in some degree, and the abrupt reperfusion inevitably leads to myocardial ischemia/reperfusion injury (MIRI). The definition of MIRI means that when myocardial cells resume perfusion after a long period of interruption of blood supply, the myocardial tissue in the ischemic area presents more serious structural damage and dysfunction than that before reperfusion [5, 6]. It is supposed to be that myocardial impairments caused by ischemia/reperfusion (I/R) can be more severe than acute myocardial infarction, and the risk of sudden cardiac death increases. However, prevention is definitely superior to treatment. Uncovering a new therapeutic approach to prevent or treat diseases for patients with a high risk of acute myocardial ischemia/reperfusion injury has become a top priority of scientists and clinicians worldwide [7, 8].

In numerous accumulating animal experiments and clinical trials, the phenomenon of ischemic tolerance induced by a series of pretreatment methods has inspired us with new ideas; that is, transient exposure to sublethal or nondestructive stimuli prior to the onset of illness can increase the tolerance of tissues or organs to the subsequent permanent and lethal injury. These pretreatment methods include ischemia (regional or remote), drug, hypoxia, and exercise, of which ischemic pretreatment (IP) has been confirmed to be one of the most effective endogenous protective mechanisms. However, in the view of applicability, the limitations and negative side effects of all the above pretreatment methods cannot be ignored, especially for weak or severely ill patients, which restricts the further clinical application.

Electroacupuncture (EA) is a novel avenue improved on traditional acupuncture therapy combined with modern electric techniques, which reflects the ideas of “treating before sick” in traditional Chinese medicine (TCM) and is well accepted as a complementary therapy throughout several Asian countries [9–11]. Furthermore, EA plays an indispensable role in treating different brain and heart diseases. Evidence has shown the potential benefits of EA pretreatment against myocardial ischemic/reperfusion injury and improving the quality of life for patients.

So far, the efficacy of EA pretreatment at Neiguan (PC6) inducing ischemic tolerance and protecting the injured myocardium has been continuously demonstrated [12]. Since EA pretreatment is simple, safe, and convenient, it is clinical applicability for prevention and not just treatment of ischemic cardiac disease. In this paper, we investigate the evidence of EA pretreatment inducing cardioprotection effect from both clinical data and experimental studies, parameters of EA pretreatment, and the intracellular pathways involved in this protection.

## 2. The Origin of EA Pretreatment

With the continuous development of medicine, modern medical needs are not only satisfied with the treatment of diseases but also require more active prevention. The definition of pretreatment was first proposed in the mid-1980s; Murry and colleagues observed the phenomenon of ischemic tolerance induced by IP in myocardial ischemia/reperfusion model of dogs; after several times of short-term repeated I/R treatment, myocardium enhanced the resistance to subsequent lethal MIRI stimulation, and myocardial damage caused by I/R such as necrosis, arrhythmia, myocardial systolic, and diastolic dysfunction was alleviated [13], which provided a new meaningful idea for preventing and treating IHD. Furthermore, studies have indicated that other pretreatment methods in modern medicine including drug, hypoxia, and exercise can also induce ischemic tolerance mimicking IP, one of the most effective endogenous protection mechanisms currently recognized [14], making a protective effect to the heart [15–18]. However, considering the perspective of clinical application and patient acceptability, these pretreatment measures still have limitations to varying degrees, especially for patients with severe illness. Therefore, the study and exploration of a rational, effective, and clinically feasible medicine therapy has become a hot spot in the field of myocardial ischemic tolerance.

Acupuncture therapy is based on “treating before sick” idea, one of the primary thinking in oriental medicine, which refers to piercing fine needle into the specific acupoints of the human body according to the meridian theory, used for the prevention, health care, and treatment of various diseases. In addition, a growing number of subhealth or sick people in Western society engaged in some form of complementary and alternative medicine (CAM) therapy, in which acupuncture therapy was the most frequently adopted. Moreover, acupuncture pretreatment consists of hand acupuncture pretreatment, moxibustion pretreatment, and EA pretreatment, and its great significance for the prevention and treatment of ischemic myocardial damage, such as arrhythmia, angina, or even sudden cardiac death in experimental studies and clinical tests has been verified [19–22].

EA is a combination treatment of conventional acupuncture and electric current with certain parameters used to stimulate acupoints and tissues instead of manual operation. In 2003, the concept of “EA pretreatment” was first proposed, suggesting that EA stimulus manipulated in advance inherited the subsequent ischemic brain injury in rats with middle cerebral artery occlusion (MCAO) model [23]. In the follow-up experiment, the efficacy of EA pretreatment generating ischemic tolerance mimicking IP has been continuously discovered in the heart. In 2014, Huang and colleagues, first explored the protective effect of EA pretreatment at Neiguan (PC6) in rats against myocardial ischemia and reperfusion injury from the perspective of gene expression [24]. In this experiment, the EA group rats were treated with electroacupuncture at the Neiguan (PC6) for 2 weeks before the model was created. And the parameter of electroacupuncture stimulation was 20 minutes per day, at a frequency of 2/15 Hz and a current intensity of 1 mA. After 24 hours of EA pretreatment, the rats were ligated with the left anterior descending coronary artery (LADCA) for 30 minutes and perfused for 240 minutes to prepare the MIRI model. As a result, the survival rate and arrhythmia score of the rats in the EA group were significantly higher than those in the I/R group, and the myocardial infarct size was significantly reduced (7.69% in the EA group versus 17% in the I/R group). In the end, the cardioprotection that EA pretreatment at Neiguan (PC6) rescued ischemic myocardium against MIRI by regulating the expression of functional genes in rats was proved.

## 3. Evidence for a Link Impact of EA Pretreatment against MIRI: Clinical Data

Although extensive experimental studies have proved the cardioprotective effect of EA pretreatment against MIRI, there is still doubt whether EA pretreatment can be used in clinical practice. Up till now, substantial evidence has shown the protective effect of acupuncture pretreatment for ischemic myocardium in accelerating the recovery of heart surgery, enhancing the left heart function, improving the symptoms of angina pectoris and palpitation, or reducing ischemia myocardial injury in patients [9, 12, 25–27].

In 2010, Yang and colleagues recruited 60 patients with acquired valvular heart disease requiring heart valve replacement, which were randomly assigned into the EA pretreatment group and the control group [12]. Five days before the operation, the EA pretreatment group received 30 minutes of electroacupuncture stimulation of bilateral Neiguan (PC6), Lieque (LU7), and Yunmen (LU2) every day as a contrast to the sham stimuli for the control group. The experimental data displayed that, compared with the control group, the level of serum cardiac troponin I (cTnI) in the EA pretreatment group decreased significantly at 6, 12, and 24 hours after the operation, the level of troponin I(TnI) decreased significantly at 6, 12, and 24 hours after aortic cross clip resection, the inotrope score decreased at 12, 24, and 48 hours after arriving in an intensive care unit (ICU), and eventually the patients' retention period in the ICU was shortened, indicating that EA pretreatment ameliorated the myocardial injury after heart valve replacement operation in adults.

In 2015, Wang and colleagues designed a 2-year follow-up randomized clinical trial and recruited 388 adult patients with stable angina, unstable angina, or asymptomatic myocardial ischemic to explore whether EA pretreatment played a role in cardioprotection for patients undergoing PCI [25]. The plan they adopted was that the EA pretreatment group was given Neiguan (PC6) and Ximen (PC4) for 30 minutes of stimulation at 1–2 hours before PCI, whereas the control group was given false electrode without electrical stimulation. The experimental data revealed that, compared with the control group, the incidence of myocardial infarction type 4a (MI4a) in the EA pretreatment group decreased significantly 24 hours after PCI, the incidence of major adverse cardiac/cerebrovascular events shown by echocardiography decreased significantly six months after the operation, and the release of cTnI in patients was reduced, suggesting that EA pretreatment protected the patients with coronary heart disease (CHD) after PCI operation against the myocardial ischemic and reperfusion injury.

## 4. Evidence for a Link Impact of EA Pretreatment against MIRI: Experimental Studies

Through delivering stimulation at specific acupoints in the body, EA pretreatment enhances the afferent impulses and improves acupuncture outcome associated with the local and systemic regulation as a candidate for the prevention or rehabilitation of ischemic heart disease. To date, a large number of animal experiments have been performed to demonstrate that EA pretreatment improves the tolerance of myocardial cells to ischemia/reperfusion stimulation, combating myocardial ischemia/reperfusion injury.

### 4.1. Effect Evaluation Parameters of EA Pretreatment

In terms of relevant evaluation parameters, the measurement of myocardial infarction area, evaluation of arrhythmia, and determination of biochemical markers in serum related to myocardial injury were often used to measure the effectiveness of electroacupuncture pretreatment against MIRI.  Table 1 summarizes the effect evaluation parameters of EA pretreatment against MIRI in animal experiments.

The effect evaluation parameters of EA pretreatment against MIRI in animal experiments are summarized. EA: electroacupuncture; MIRI: myocardial ischemia/reperfusion injury; LADCA: left anterior descending coronary artery; ECG: electrocardiogram; [Ca^2+^]_*i*_: free calcium concentration; Cx_43_: gap junction protein 43; SOD: superoxide dismutase; MDA: malondialdehyde; Nrf2: nuclear factor erythroid-2-related factor 2; HO-1: heme oxygenase-1; NO: nitric oxide; NOS: nitric oxide synthase.

### 4.2. Intervention Parameters of EA Pretreatment

EA pretreatment has some different characteristics from other pretreatment methods, such as the specific intervention parameters of electrical stimulation and particular acupoints selected. However, the electroacupuncture pretreatment scheme adopted in various studies is not the same. It has been found that EA pretreatment at Neiguan (PC6) for 1 d, 3 d, 7 d, and 12 d has shown good myocardial protective effect [24, 28, 31]. In TCM, Neiguan (PC6) is a critical acupoint selected for acupuncture in the treatment of many diseases and has a strong correlation and specificity with the heart. Therefore, Neiguan (PC6) acupoint is the first choice by investigators in the relevant studies of EA pretreatment against myocardial ischemia/reperfusion injury [32].  Table 2 summarizes the intervention parameters of EA pretreatment against MIRI in animal experiments.

The electrical stimulation parameters, acupoints, and potential protective mechanisms of EA pretreatment against MIRI in animal experiments are summarized. EA: electroacupuncture; MIRI: myocardial ischemia/reperfusion injury; ESP: electrical stimulation parameters; ECM: extracellular matrix; MAPK: mitogen-activated protein kinase; SGIR: simulative global ischemia reperfusion; [Ca^2+^]_i_: free calcium concentration; Cx_43_: gap junction protein 43; Nrf 2-ARE: NF-E2-related factor 2 antioxidant response element; ET: endothelia; CK: creatine kinase; HSP: heat shock protein.

### 4.3. Proposed Myocardial Protection Mechanisms of EA Pretreatment

**Figure 1:**
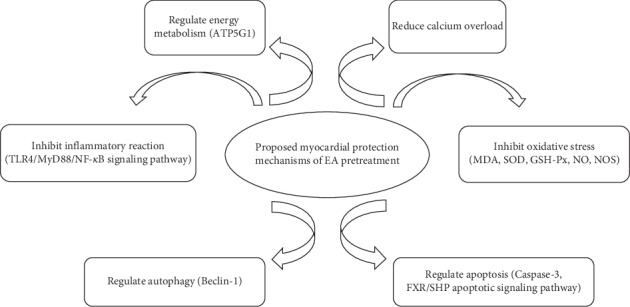
Proposed myocardial protection mechanisms of EA. EA: electroacupuncture; ATP5G1: ATP synthase F(0) complex subunit C1; TLR4: toll-like receptor 4; MyD88: myeloid differentiation primary response gene 88; NF-*κ*B: nuclear factor kappa-B; MDA: malondialdehyde; SOD: superoxide dismutase; GSH-Px: glutathione peroxidase; NO: nitric oxide; NOS: nitric oxide synthase; FXR: farnesoid X receptor; SHP: small heterodimer partner.

At present, the molecular mechanisms involved in MIRI include the adhesion of neutrophils and vascular endothelial cells, production of oxygen free radicals, calcium overload, abnormal energy metabolism, cardiomyocyte apoptosis, and vasospasm [33]. In animal studies, it was found that the pretreatment of Neiguan (PC6) with EA could protect the heart against ischemia and reperfusion injury, such as regulating the contraction of cardiac muscle and vascular smooth muscle by regulating the inflammatory immune response and apoptosis pathway [24]. Proposed protective mechanisms of EA pretreatment were summarized in Figure 1.

#### 4.3.1. EA Pretreatment Regulates Energy Metabolism

One leading reason inducing myocardial ischemia/reperfusion injury is the deficiency of myocardial energy synthesis in the ischemic area, of which the pathological basis includes dysfunction of ATP synthase F(0) complex subunit G1 (ATP5G1) [34]. A large amount of reactive oxygen species (ROS) due to excess hypoxanthine is one of the classic pathological mechanisms of ischemia/reperfusion injury, which is active in nature, contributes to mitochondrial membrane peroxidation and permeability enhancement, and eventually gives rise to mitochondrial swelling and rupture. As acting on the inner membrane of mitochondria, ROS further affects the function of ATP synthase, the ATP synthesis, and the energy supply of cardiomyocytes [35]. Chen and colleagues observed that the EA pretreatment of Neiguan (PC6) defended the structure and function of mitochondria against I/R stimulus, prevented myocardial tissue structure to be destroyed, and repaired myocardial ischemic injury in rats [36].

#### 4.3.2. EA Pretreatment Inhibits Inflammation Reaction

Modern medical research proves that the inflammation reaction triggered by ischemia/reperfusion stimulation may cause secondary damage for ischemia tissues or organs [37, 38]. One of the primary signs of ischemia/reperfusion injury is the inflammatory response in the myocardial risk area and microvascular complications induced by the infiltration of inflammatory leukocytes released by cytokines [39]. In fact, an excessive inflammatory response will not be conducive to the survival of myocardial cells and may lead to the reconstruction of the left ventricle. The fact proved that acupuncture played a good anti-inflammatory role in regulating the inflammatory response pathway, cholinergic anti-inflammatory pathway, and inflammatory factors. EA pretreatment can reduce the content of inflammatory cytokines and alleviate oxidative stress response in diabetic rats, producing protection effect to the heart suffering from ischemia and reperfusion injury [40]. Zhu and colleagues reported that EA pretreatment at Neiguan (PC6) alleviated myocardial ischemia/reperfusion injury by regulating toll-like receptor 4 (TLR4)/myeloid differentiation primary response gene 88 (MyD88)/nuclear factor kappa-B (NF-*κ*B) signal pathway in rats [41].

#### 4.3.3. EA Pretreatment Reduces Calcium Overload

The dynamic balance of Ca^2+^ in cardiomyocytes significantly influences the systolic function of the heart. Once the myocardial cell ischemia events occur, ATP supplying cell metabolism demand will be obviously insufficient, and Ca^2+^ transport in the endoplasmic reticulum can be abnormal, which contributes to calcium overload of cardiomyocytes and aggravates the myocardial cell injury [42, 43]. During the occurrence and progression of myocardial ischemia/reperfusion injury, myocardial cell damage induced by calcium overload results in cardiac contraction disorders and ventricular remodeling. Han and colleagues reported that EA pretreatment maintained calcium homeostasis and enhanced oxygen free radical scavenging capability of cardiomyocytes in diabetic rats against ischemia and reperfusion injury [44].

#### 4.3.4. EA Pretreatment Inhibits Oxidative Stress

When myocardial ischemia/reperfusion injury event occurs, oxidative stress will cause a large number of oxygen free radicals to be released, which is considered to be an important factor for the disruption of heart function, as well as the main factor leading to myocarditis and extracellular remodeling [8, 45]. Evidence shows that EA pretreatment effectively inhibits the expression of malondialdehyde (MDA) and promotes the expression of superoxide dismutase (SOD) in myocardial cells of acute myocardial ischemia/reperfusion injury rats, and the mechanism involves activating endogenous antioxidant pathway, improving the ability to scavenge oxygen free radicals, and reducing the damage of lipid peroxide [29]. Zhang and colleagues observed that EA at Neiguan (PC6) effectively relieved lipid peroxidation reaction of the myocardial cell membrane and activated oxygen free radical scavenging system, such as regulating the content of peroxidase glutathione peroxidase (GSH-Px) and MDA to enhance myocardial cells' resistance for ischemia and reperfusion injury [9]. Moreover, other experimental data also indicated that the mechanism of EA at Neiguan (PC6) against myocardial ischemia/reperfusion injury may be relevant to the upregulation of nitric oxide (NO) and nitric oxide synthase (NOS) contents in rat myocardium [46].

#### 4.3.5. EA Pretreatment Regulates Autophagy

Cardiomyocytes show strong autophagy characteristics and autophagy plays an important role in myocardial ischemia/reperfusion injury [47, 48]. Autophagy is a process in which autophagy bodies phagocytize their own organelles to synthesize energy and ease the hunger of the body. However, autophagy is two-sided. As one of the physiological processes, autophagy has an important effect on cells to maintain self-stability and survival in the physiological and stress condition, but in the pathological condition, excessive autophagy can cause cell autophagic death [49–52]. Studies show that myocardial cell death caused by myocardial ischemia/reperfusion injury can be attenuated by autophagy regulated [53]. Tan and colleagues demonstrated that EA pretreatment reduced myocardial cell damage by regulating autophagy of cardiomyocytes and downregulating the expression level of beclin-1 protein when myocardial ischemia/reperfusion injury happened [54]. EA pretreatment at Neiguan (PC6) improved the autophagy level and reduced the apoptosis reaction of myocardial ischemia cells in rats, of which the cardioprotection effect produced was similar to IP [55]. Chen and colleagues observed that EA treatment before ischemia for 1–5 days all reduced the myocardial infarction area in MIRI rats and the mechanism was supposed to be related to the decreased autophagy level of myocardial cells [56].

#### 4.3.6. EA Pretreatment Reduces Apoptosis

It has been demonstrated that apoptosis is the ultimate ending caused by myocardial ischemia/reperfusion injury, initiated at the time of myocardial ischemia beginning, amplified, and involved in the whole myocardial death during reperfusion [57]. It was reported that EA pretreatment could decrease myocardial ischemia/reperfusion infarction size and improve cardiac function via the inhibition of the occurrence of myocardial cell apoptosis [58]. Zhou and colleagues found that EA pretreatment inhibited myocardial cells apoptosis by downregulating the expression level of caspase-3 protein in rats suffering from myocardial ischemia/reperfusion injury [59]. Furthermore, EA pretreatment rescued the ischemia myocardium and improved the cardiac function in MIRI model rats by regulating the farnesoid *X* receptor (FXR)/small heterodimer partner (SHP) cell apoptosis signal pathway and related gene expression [60]. Zhong and colleagues indicated that, by means of reducing cell autophagy and cell apoptosis, EA treatment for 30 minutes let the infarction area obviously be reduced in rats suffering from myocardial ischemia/reperfusion injury [61].

#### 4.3.7. Other Mechanisms

It was reported that EA at Neiguan (PC6) alleviated the further injury of damaged myocardium after ischemia/reperfusion, and the mechanism was relative to the inhibition of cardiac sympathetic activation with protein kinase C (PKC) pathway, noradrenaline release, and the activity of cardiac sympathetic nerve partially [62, 63]. In addition, Gao and colleagues pretreated myocardial I/R model rats with EA for 3 consecutive days and EA pretreatment showed the beneficial cardioprotection effect by the reason of significantly inhibiting the overexpression of *β*1-adrenoceptor protein [63]. Yang and colleagues pretreated MIRI rabbits with EA and moxibustion at Neiguan point (PC6) for 5 days and found that the content of adenosine in the serum of animals increased compared with other groups [30]. In another experiment, Wang and colleagues pretreated New Zealand white rabbits with EA for 5 days, then made the MIRI model by coronary artery ligation at 24 and 48 hours, and found that, compared with the model group, the content of plasma endothelin in the EA pretreatment group decreased significantly, further explaining the protective effect of EA pretreatment on myocardium [64]. In addition to this, it was found that the dephosphorylating of Connexin (Cx) induced by myocardial ischemia weakened the coupling between cells and caused serious arrhythmia, which could be improved by EA pretreatment significantly [28, 65, 66].

## 5. Conclusion

Acupuncture, one of the classical therapies in Chinese medicine throughout history, has been used to prevent and treat diseases for thousands of years. Currently, the existing evidence has fully demonstrated that EA pretreatment can alleviate ischemic myocardial injury and promote the recovery of heart function. Similar to other preconditioning procedures, EA pretreatment can induce ischemia tolerance against myocardial ischemia/reperfusion injury. However, different from other preconditioning procedures, the cardioprotection effect induced by EA pretreatment has to do with the specific electrical stimulation parameters and acupoint selected, of which multichannel, multilevel, and multitarget mechanisms are thought to be involved in the molecular mechanism.

In basic researches and clinical tests, the investigators have observed the intensive changes of biochemical, morphological, and gene targets in the myocardial cells after I/R injury, of which EA pretreatment is suggested to be the reason. Nevertheless, little work focuses on the reason and detailed information about the mechanisms of EA pretreatment has not yet fully understood. For example, EA pretreatment has been shown to regulate the TLR4/MyD88/NF-*κ*B signal pathway and inhibit the overoccurrence of the inflammatory reaction, thereby combating the myocardial injury after I/R in rats. But, it is still not clear how EA treatment before ischemia brings about the change of inflammation-related factors after reperfusion. What occurs during the process of EA pretreatment still needs to be explained.

In addition, compared with other preconditioning procedures, EA pretreatment possesses some unmatched merits, such as low cost, safety, and easy operation, which is more easily accepted by patients in the clinic. For example, although IP is currently recognized as one of the most effective endogenous protective mechanisms, the protective effect is affected by factors such as age, hypertension, diabetes, and drugs (such as propofol). Moreover, acute cardiovascular events are usually unpredictable, and all these reasons limit the application of IP clinically. Similarly, there is still a gap between the potential benefits of EA and practical application in the prevention of myocardial ischemia/reperfusion injury. EA pretreatment has good repeatability and stability, of which the benefits are tightly associated with the determination of parameters and acupoints carried out. However, although Neiguan (PC6) acupoint is the first choice of selection in most studies currently, the parameters of EA pretreatment have not yet been unified completely, such as stimulation frequency, current intensity, pulse width, pulse interval, and intervention time. In the course of searching the literature, we found that in some experiments the specific scheme of EA pretreatment carried out was not full. Much more meticulous researches on EA pretreatment antagonizing myocardial ischemia/reperfusion injury need to be conducted for exploring the best parameters to achieve the best outcome. As for the possibility of clinical application of EA pretreatment in the future, it is necessary to develop preischemic markers indicating the effectiveness of EA pretreatment, with a hope for applying individual EA pretreatment according to genders, age, and pathological states such as hypertension. At last, we need more data for supporting the cardioprotective effect of EA pretreatment. Thus, large sample and multicenter randomized control trials need to be performed, which may provide more powerful evidence for making the cardioprotective mechanism of EA pretreatment and further clinical practice clear. Although there is still lots of work to be done, if we can successfully find satisfactory answers to the above questions, we can use EA pretreatment to turn the wish of patients with a high risk of acute myocardial ischemia/reperfusion injury to live a better life into reality.

## Figures and Tables

**Table 1:** Summary of effect evaluation parameters of EA pretreatment against MIRI in animal experiments.

Reference	Species	Models	Infarct reduction	Effect evaluation parameters
Gao et al. [22]	Rats	30 min LADCA ligation, 15 min reperfusion	∼44%	ST segment of ECG; cardiac arrhythmias; risk zone/infarct size
Huang et al. [24]	Rats	30 min LADCA ligation, 240 min reperfusion	∼55%	Arrhythmic; infarct size and myocardial enzymes; gene expressions genome-widely
Gao et al. [28]	Rats	40 min isolated heart reduced-flow perfusion, 10 min reperfusion	No morphological study	Arrhythmia; [Ca^2+^]_i_ in resting single ventricular myocyte; Cx_43_ protein levels in ventricular myocytes; nonphosphorylated Cx_43_ in ventricular myocytes
Shao et al. [29]	Rats	30 min LADCA ligation, 30 min reperfusion	No morphological study	ST segment displacement value; SOD, MDA content in serum; cardiomyocyte tissue structure; ultramicrostructure of cardiomyocytes; expression of Nrf2 and HO-1 genes in myocardial tissue
Wang et al. [30]	Rabbits	40 min LADCA ligation, 60 min reperfusion	No morphological study	NO, NOS content in serum; adenosine content in serum

**Table 2:** Summary of intervention parameters of EA pretreatment against MIRI in animal experiments.

Reference	EA pretreatment		Mechanisms
	ESP	Acupoint	
Gao et al., 2006 [22]	20 Hz, 5 mA, 30 min, 3 days	Neiguan acupoint (PC6)	*β*-Adrenoceptors was involved
Huang et al., 2014 [24]	2/15 Hz, 1 mA, 20 min, 12 days, a day of rest after six days	Neiguan acupoint (PC6)	These genes were involved in multiple pathways, including ECM, MAPK signaling, apoptosis, cytokine, and leukocyte pathways; in addition, some pathways were uniquely regulated by EA, such as oxidative stress, cardiac muscle contraction, gap junction, vascular smooth muscle contraction, hypertrophic, NOD-like receptor, P53, and B-cell receptor pathways
Gao et al.,			
2015 [28]	20 Hz, 1–3 mA, 30 min, 3 days	Neiguan acupoint (PC6)	EA pretreatment could inhibit SGIR-induced calcium overload and [Ca^2+^]_i_ oscillations, reduce nonphosphorylated Cx_43_, and enhance the corresponding phosphorylated Cx_43_ in the cardiac cells
Shao et al., 2017 [29]	2–100 Hz, 1 mA, 30 min, 7 days	Jiaji T4∼T5 acupoint (EX-B2), Neiguan acupoint (PC 6), and Quchi acupoint (LI 11)	Acupuncture pretreatment at “Jiaji” (EX-B2) had the protective effect on MIRI, which was probably relevant with the upregulation of Nrf 2-ARE pathway expression, the activation of the endogenous antioxidative pathway, the improvement of oxygen free radical scavenging capacity, and the alleviation of lipid peroxide damage
Wang et al., 2014 [30]	20 min, 5 days	Neiguan acupoint (PC6)	Acupuncture and moxibustion pretreatment may suppress MIRI-induced increase of plasma ET and serum CK and upregulate myocardial HSP 70 protein expression in MIRI rabbits
